# Nanotechnology in Agriculture: Manganese Ferrite Nanoparticles as a Micronutrient Fertilizer for Wheat

**DOI:** 10.3390/plants13101395

**Published:** 2024-05-17

**Authors:** Xiwei Huang, Xin Wang, Xingxing Liu, Liping Cheng, Jianqing Pan, Xiaoe Yang

**Affiliations:** 1 Ministry of Education (MOE) Key Laboratory of Environmental Remediation and Ecosystem Health, College of Environmental and Resources Sciences, Zhejiang University, Hangzhou 310058, China; 22114147@zju.edu.cn (X.H.); 22214128@zju.edu.cn (X.W.); xxlliu@zju.edu.cn (X.L.); lpcheng@zju.edu.cn (L.C.); 2 Agriculture Bureau of Changxing County, Huzhou 323000, China; zjcxpjq@126.com

**Keywords:** MnFe_2_O_4_ nanoparticles, wheat production, biofortification, foliar application, nano-fertilizer safety

## Abstract

Limited research has focused on nanoparticle (NP) applications’ impact on edible wheat parts in a field environment. Here, we studied the nutritional quality of edible parts of wheat (*Triticum aestivum* L.) with a field experiment by spraying MnFe_2_O_4_ nanoparticles. Wheat was foliar sprayed with 0, 25, 50, and 100 mg/L composite manganese ferrite (MnFe_2_O_4_) NPs during 220 d of a growth period. Ionic controls were prepared using the conventional counterparts (MnSO_4_·H_2_O and FeSO_4_·7H_2_O) to compare with the 100 mg/L MnFe_2_O_4_ NPs. After three consecutive foliar applications, nanoparticles demonstrated a substantial elevation in grain yield and harvest index, exhibiting a noteworthy increase to 5.0 ± 0.12 t/ha and 0.46 ± 0.001 in the 100 mg/L NP dose, respectively, concomitant with a 14% enhancement in the grain number per spike. Fe, Mn, and Ca content in grain increased to 77 ± 2.7 mg/kg, 119 ± 2.8 mg/kg, and 0.32 ± 7.9 g/kg in the 100 mg/L NPs, respectively. Compared to the ion treatment, the 100 mg/L NP treatments notably boosts wheat grain crude protein content (from 13 ± 0.79% to 15 ± 0.58%) and effectively lowers PA/Fe levels (from 11 ± 0.7 to 9.3 ± 0.5), thereby improving Fe bioavailability. The VSM results exhibited a slight superparamagnetic behavior, whereas the grains and stems exhibited diamagnetic behavior. The results indicate that the nanomaterial did not accumulate in the grains, suggesting its suitability as an Fe and Mn-rich fertilizer in agriculture. Above all, the foliar application of nanocomposites increased the concentrations of Fe, Mn, and Ca in wheat grains, accompanied by a significant enhancement in grain yield. Therefore, the research results indicate that the foliar application of MnFe_2_O_4_ NPs can positively regulate wheat grains’ nutritional quality and yield.

## 1. Introduction

Wheat is widely planted throughout the world. In northern China, wheat grains contribute to more than 40% of the protein required by the human body [1]. Historically, increasing wheat yields has been focused on, with less attention given to enhancing the micronutrients of edible crop parts [2,3]. In developing countries, many children suffer from hidden hunger, lacking specific vitamins and micronutrients such as Fe, Zn, and vitamin A [4,5]. The hidden hunger is associated with the monotonous dietary structure and imbalanced nutritional intake prevalent in low-income nations. In these areas, individuals often rely on inexpensive staples like bread made from wheat flour for sustenance without access to a variety of foods to fulfill their nutritional needs [6]. Hence, there is an urgent need to enhance the micronutrient content such as Fe and nutrition in the grain of edible plants to prevent nutritional deficiencies among individuals. People aim to improve crops’ nutritional composition by applying chemical fertilizers. Chemical agricultural products are essential for crop growth; however, fertilizers have been misused and overused [7,8]. Therefore, researchers focus on enhancing plant nutrient uptake efficiency by refining the chemical fertilizers. Recently, studies have shown that under equivalent ionic concentrations, nano-fertilizers composed of macronutrients or micronutrients exhibit higher efficiency than traditional fertilizers and demonstrate the effective reduction of nutrient leaching [9,10].

Nanomaterials hold significant promise regarding their application in agricultural scenarios due to their tunable pore size, high surface-to-volume ratio, and physicochemical properties. They are categorized into three groups: nanoparticle-containing macronutrients, nanoparticle-containing micronutrients, and nanoparticles as nutrient carriers [11]. Known applications of nanotechnology in agriculture include nano-sensors, nanozymes, delivery of pesticides, and environmental applications. Considering prevailing market trends and recent studies, nanotechnology is anticipated to assume a progressively prominent role in the field of agriculture [7,12,13,14,15,16]. Studies have confirmed that nanomaterials can enter plants through both root and leaf stomata [17,18,19,20]. Utilizing the plant root absorption characteristics for nutrient supplementation, soil fertilization remains the most commonly employed method. However, it is susceptible to various factors such as pH, root microbiota, and exudates [21]. Applying nanoparticles to the root system blocks the microstructures on the surface of root hairs, thereby hindering the absorption and transport of water and nutrients by the roots, affecting the growth and development of crops [22]. However, foliar fertilization is an effective way to provide the required micronutrients for plants [23]. Between these two application approaches, foliar spray outperforms soil application in terms of both nutrient uptake and delivery efficiency [12]. Compared with soil treatment, spraying nano-scale Mn could provide superior control over plant responses and enhance grain yield [24]. Foliar applications of carbon dots resulted in a greater increase in nutrient elements such as Fe and Mn in wheat grains compared to soil irrigation [25]. The foliar spray application of CeO_2_ NPs was more beneficial for *Phaseolus vulgaris* L. growth compared to soil application, as evidenced by proteomics and metabolomics [26].

Applying the same dosage of nano-fertilizers exhibits diminished harm to crops in comparison to conventional chemical fertilizers [27,28]. Gao et al. discovered that metallic nanoparticles often exhibited lower toxicity to plants than their corresponding ionic counterparts at equipotent concentrations [29]. In the study of Fe_2_O_3_ NPs and Fe^3+^ fertilizer applications on citrus plants, Hu et al. observed that both fertilizers promoted chlorophyll synthesis in plants at a concentration of 100 mg/L [30]. However, Fe^3+^ fertilizer exhibited higher toxicity in the plants. Results from experiments on wheat by Baddar et al. [31] lend support to this perspective in the case of both Zn nanoparticles and ZnSO_4_ treatment. These studies contribute to our understanding of the effects of nanoparticles and ion fertilizers on plant growth, but the current research predominantly focuses on controlled environments such as laboratories, greenhouse hydroponics, or potted experiments. Under natural conditions, the impact of spraying nanoparticles on the quality of edible parts in crops requires further investigation. Additionally, the absence of actual agricultural production experiments hinders a comprehensive assessment of the practical effects of nanoparticles.

Importantly, since Fe and Mn are involved in photosynthesis and play a major role in promoting plant growth, there is a special need to study the effect of composite manganese ferrite applications on plant growth promotion. Simultaneously, the utilization of MnFe_2_O_4_ as a nano-fertilizer for increasing crop yield and nutrients has garnered interest recently [32]. Reportedly, binary nanometal oxides outperform single-metal oxides [13]. Hence, exploring the advantageous outcomes of binary nanometal oxides in promoting plant growth holds significant research importance. Here, we hypothesize that the application of nano-fertilizers can enhance wheat chlorophyll content and improve wheat yield, biomass, and nutritional elements such as Fe and Mn in the edible parts. The investigation includes: (1) examining the impact of MnFe NPs on wheat growth and photosynthesis, (2) assessing the influence of NPs on the concentrations of Fe and Mn in wheat tissues, and (3) researching the nutrients in the edible parts. These studies aim to enhance our understanding of the potential and limitations of nano-enabled strategies for improving crop productivity and nutritional quality.

## 2. Results and Discussion

### 2.1. Nanocomposite Characterization of MnFe_2_O_4_ NPs

**Figure 1:**
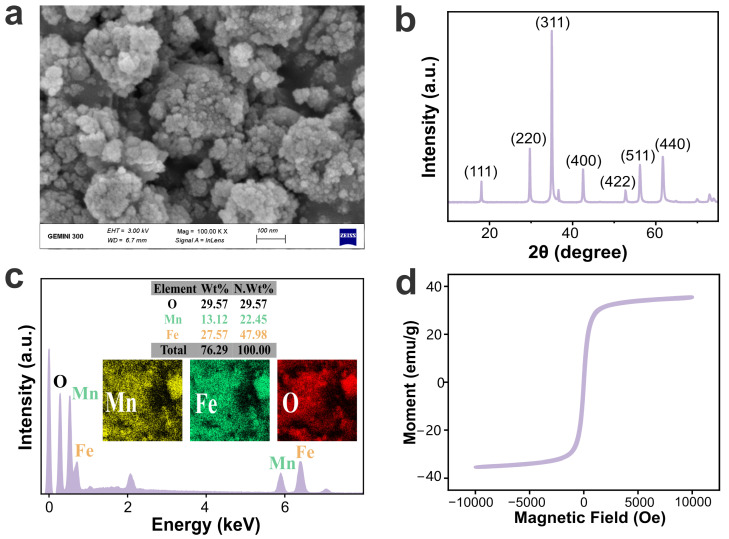
Characterization of MnFe_2_O_4_ nanoparticles (NPs). (**a**) Scanning electron microscopy (SEM) analysis of MnFe_2_O_4_ NPs; (**b**) X-ray powder diffraction (XRD) analysis of MnFe_2_O_4_ NPs; (**c**) X-ray spectrometer (EDX) analysis of MnFe_2_O_4_ NPs; (**d**) vibrating sample magnetometer (VSM) curves of MnFe_2_O_4_ NPs.

The irregular shapes and the crystallographic structure of magnetite nanoparticles are shown in Figure 1a and Figure 1b, respectively. SEM images showed particle aggregates, possibly due to magnetic interactions and van der Waals forces. The diffraction peaks shown in the X-ray powder diffraction (XRD) pattern align with the standard MnFe_2_O_4_ card (JCPDS card No. 10e0319) [14]. The chemical element and purity of the particles are shown in Figure 1c. The Fe, Mn, and O elements are highly concentrated in MnFe_2_O_4_ samples, and their atomic concentrations are 27.57%, 13.12%, and 59.31%, respectively. Yue found the Fe and Mn ions in the synthesized MnFe_2_O_4_ NPs were mostly in the 2+ state [32], which possibly made the Fe and Mn ions unstable and released them slowly [33]. The presence of superparamagnetic behavior with a saturation magnetization M_s_ ~35 emu/g was shown in the VSM image (Figure 1d). In reality, the variations in synthesis methods and operational parameters can influence the characteristics of MnFe_2_O_4_ NPs [34]. Relative research confirms that MnFe_2_O_4_ NPs have higher crystallinity and purity at the reaction temperature of 200 °C [35].

### 2.2. MnFe_2_O_4_ NP Distribution in Plant Tissues

**Figure 2:**
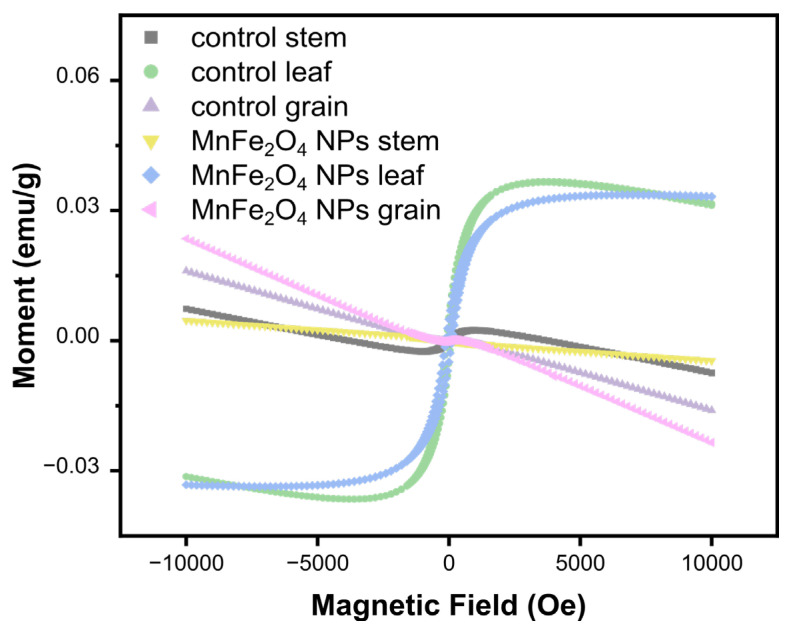
The vibrating sample magnetometer (VSM) curves of wheat leaf, stem, and grain samples under different treatments.

To assess the uptake and translocation of nanoparticles by plants, we measured the magnetization levels in plant tissues (leaves, straws, and grains). The treated leaves exhibit weak superparamagnetic behavior, while the diamagnetic behavior is observed in all the straw and grain samples (Figure 2). These results were similar to those in a study of MnFe_2_O_4_ NPs in tomatoes [32]. Typically, saturated magnetization curves are measured in magnetic materials, which can be magnetized and reach saturation under the influence of an external magnetic field. However, the control leaves showed a weak magnetic response, which was probably attributed to the influence of other biominerals or biomagnetic structures within the plant. In conclusion, the utilization of Fe NPs in wheat spray did not result in the presence of nanoparticles in the wheat grain.

### 2.3. Increased Yield of Wheat Grain upon MnFe_2_O_4_ NP Exposure

**Figure 3:**
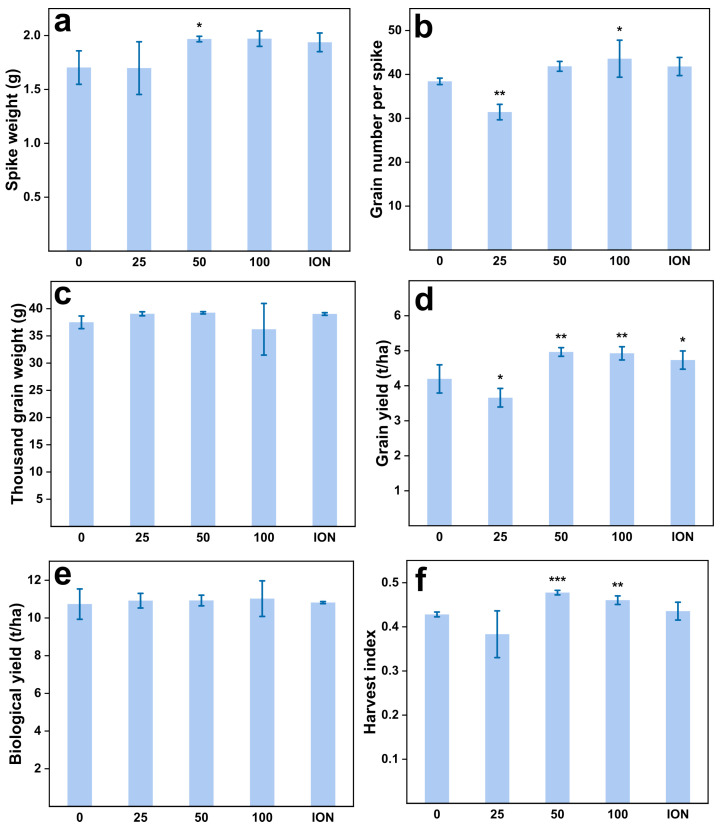
Agronomic parameters of wheat. (**a**) Spike dry weight, (**b**) grain number per spike, (**c**) thousand-grain weight, (**d**) grain yield, (**e**) biological yield, and (**f**) harvest index (HI) under the foliar application in the field experiment. Abbreviations: “0, 25, 50, 100” means the concentration of the MnFe_2_O_4_ NP application (mg/L); ION means the ionic treatments. * represents the statistically significant at *p* < 0.05, ** shows the statistically significant at *p* < 0.01, while *** shows the statistically significant at *p* < 0.001 between the control and NP treatments.

Previous studies have demonstrated that the content of chlorophyll in wheat increased due to the nanoparticle application [36,37,38]. Consistent with this, our research results demonstrated a significant (*p* < 0.05) rise in the levels of pigment after application (Figure S1). The grain yield significantly (*p* < 0.01) increased by 18% (from 4.2 ± 0.4 to 5.0 ± 0.12 mg/kg) and 17% (from 4.2 ± 0.4 to 4.9 ± 0.19 mg/kg) under the 50 and 100 mg/L treatments, respectively. The counterpart fertilizer treatments (ION) were increased by 13% (from 4.2 ± 0.4 to 4.7 ± 0.26) (Figure 3d). Those results show the possibility that nanoparticles can stimulate the expression of plant-related genes [10]. For instance, the application of chitosan nanoparticles on wheat induces the expression of auxin-related genes, expedites the biosynthesis and transportation of indoleacetic acid (IAA), and elevates IAA concentrations in wheat tissues, thereby exerting a positive impact on plant growth [39]. The second possibility is the increase in iron (Fe) content. Fe is an essential element in the synthesis of chlorophyll, and an increase in iron content corresponds to a rise in chlorophyll levels.

There were no significant changes in the spike weight, grain number per spike, thousand-grain weight, or biological yield between 100 mg/L and the control (CK) or the ion group (ION) (Figure 3). The magnitude of the harvest index (HI) was commonly used to assess the economic yield of crops [40], and the HI was significantly (*p* < 0.05) improved at 50 and 100 mg/kg (from 0.43 ± 0.005 to 0.48 ± 0.005, 0.47 ± 0.01). However, other studies found that zerovalent iron nanoparticles had no effect on cucumber biomass [41].

### 2.4. Enhanced Wheat Grain Quality by MnFe_2_O_4_ NPs

**Figure 4:**
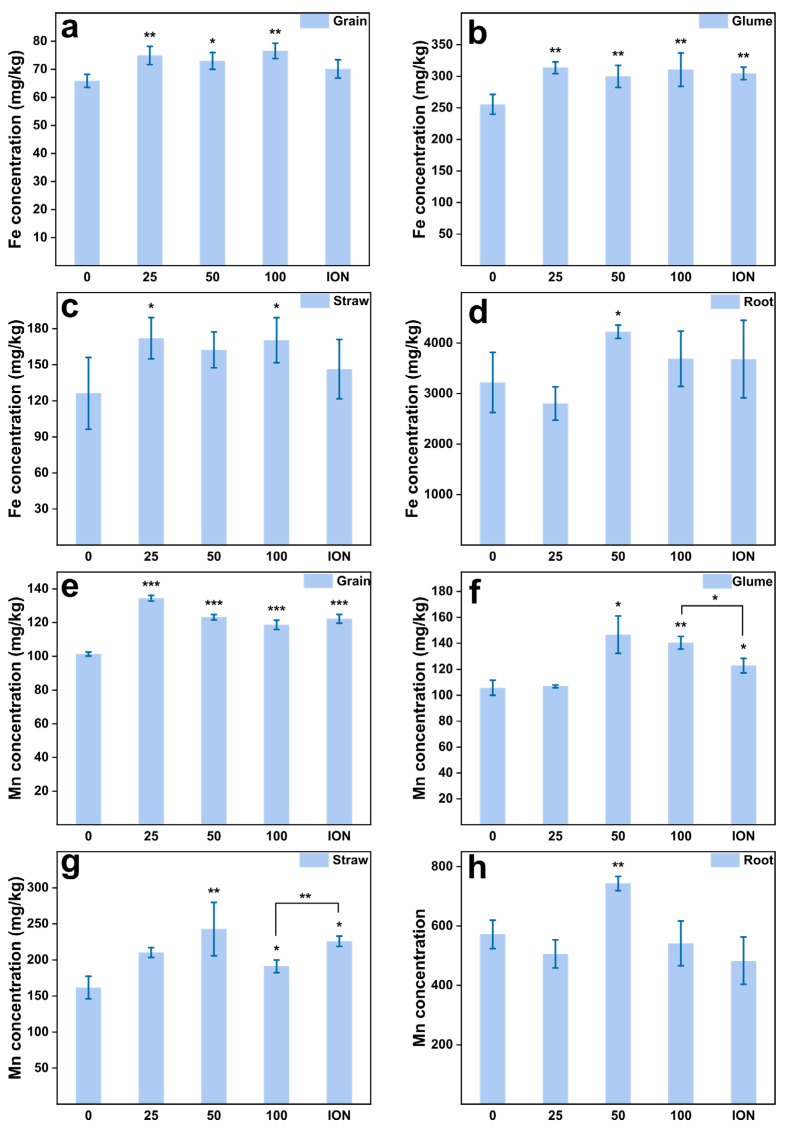
Fe and Mn concentration in different wheat tissues. (**a**) Fe concentration in grain, (**b**) Fe concentration in glume, (**c**) Fe concentration in straw, (**d**) Fe concentration in root, (**e**) Mn concentration in grain, (**f**) Mn concentration in glume, (**g**) Mn concentration in straw, (**h**) Mn concentration in root under the foliar application in the field experiment. Abbreviations: “0, 25, 50, 100” means the concentration of the MnFe_2_O_4_ NPs application (mg/L); ION means the ionic treatments. Asterisk bracket means difference between the 100 mg/L NP treatment and the ionic treatment. * represents statistically significant at *p* < 0.05, ** shows statistically significant at *p* < 0.01, while *** shows statistically significant at *p* < 0.001 between the control and NP treatments.

**Figure 5:**
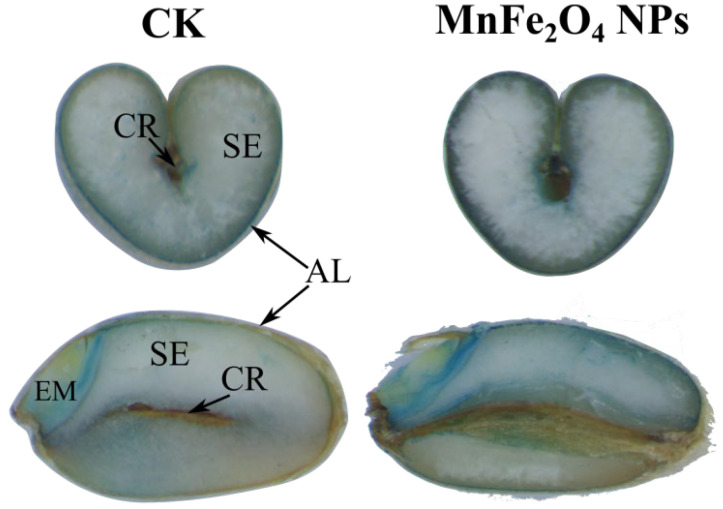
Localization of Fe by Prussian blue in the mature grains from the control plant and the treated plant. EM: embryo; SE: starchy endosperm; CR: crease; AL: aleurone layer.

To further assess the impact of nanoparticles on wheat, we measured the contents of nutrient elements (Fe, Mn, Ca, Mg, Cu, Zn, P, S) in various parts of the wheat plant. As expected, the content of Fe and Mn in the grains was significantly (*p* < 0.01) upregulated in all MnFe_2_O_4_ NP treatments (from 25 to 100 mg/L) compared to control (Figure 4a). The Fe concentration in the grains treated with 100 mg/L MnFe_2_O_4_ NPs had the highest value (76.5 ± 2.7 mg/kg) compared with the controls (65.9 ± 2.3 mg/kg). The results from Perls’ Prussian blue staining also indicated a clear improvement in iron accumulation within wheat grains (Figure 5). Earlier, it was reported that micronutrient application as a foliar spray can increase Fe content in wheat grain [42,43]. The concentration of Mn in the grain increased with MnFe_2_O_4_ NPs and observed the highest value (134.4 ± 1.6 mg/kg) at the spraying concentration of 25 mg/L. Subsequently, at higher NP applications (50 and 100 mg/L), the Mn content decreased (Figure 4e). Compared to the CK (0 mg/L), there was a remarkable increase in Fe and Mn concentrations in the wheat glume (Figure 4b,f). The Fe concentrations showed a significant increase (*p* < 0.01) in both the NP treatments ranging from 25 to 100 mg/L doses and the ion treatment (Figure 4b); Fe concentration exhibits a range of 256 ± 16 to 314 ± 9 mg/kg. The Mn concentration in the glume significantly increased in the 50 and 100 mg/L NPs as well as in the ion fertilizer (Figure 4f). Contrary to the alterations in Fe content within the glume, the concentration of Mn was higher (*p* < 0.05) in the 100 mg/L NPs dose than in the ion group. In straw, both Fe and Mn concentrations changed, significantly increasing under the treatment of 100 mg/L. The Fe and Mn contents in the roots peaked at the concentration of 50 mg/L, reaching 4225 ± 131 mg/kg and 45 ± 1.7 mg/kg, respectively.

**Figure 6:**
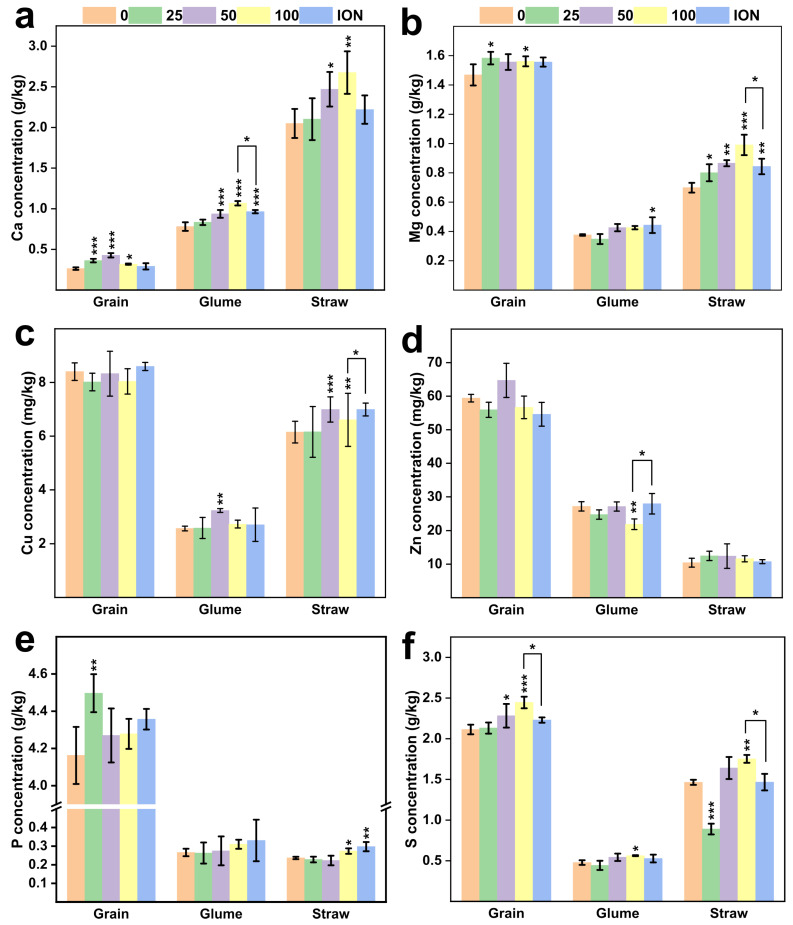
Element concentration in different wheat tissues. (**a**) Ca concentration, (**b**) Mg concentration, (**c**) Cu concentration, (**d**) Zn concentration, (**e**) P concentration, (**f**) S concentration in different wheat tissues (grain, glume, straw) under the foliar application in the field experiment. Abbreviations: “0, 25, 50, 100” means the concentration of the MnFe_2_O_4_ NP application (mg/L); ION means the ionic treatments. Asterisk bracket means difference between the 100 mg/L NP treatment and the ionic treatment. * represents statistically significant at *p* < 0.05, ** shows statistically significant at *p* < 0.01, while *** shows statistically significant at *p* < 0.001 between the control and NP treatments.

More interestingly, the application of nanomaterials can increase the content of other elements in grains. At the 100 mg/L NP dose, Ca, Mg, and S contents in the grains showed a significant increase (*p* < 0.05) (Figure 6). The foliar application of Ca at concentrations of 25, 50, and 100 mg/L resulted in a notable enhancement (0.36 ± 0.02, 0.43 ± 0.03, 0.32 ± 0.01 g/kg), particularly at 50 mg/L, where there was a 62 percent (from 0.26 ± 0.02 to 0.43 ± 0.03 g/kg) increase. However, at the 100 mg/L concentration, the Ca content declined (Figure 6a). This suggests that the foliar application of nanomaterials at lower concentrations can effectively promote Ca absorption in wheat, thereby achieving a positive effect on calcium augmentation. As the application dose increased, the Ca content in the glumes correspondingly rose (Figure 6a). The wheat sprayed with 100 mg/L NPs had the highest Ca value in glume (37% and 10% more than control and ion treatment, respectively). The Mg concentration in the grain was notably higher (*p* < 0.05) at 100 mg/L compared to the control, and with increasing application doses, the Mg content in the straw also gradually increased (Figure 6b). Thus, the application of trace element nano-fertilizers not only enhances the content of these elements in crops and promotes plant growth but also increases the variety of available nutrient elements for plant consumers.

### 2.5. Decreased Phytic Acid by MnFe_2_O_4_ NPs

**Figure 7:**
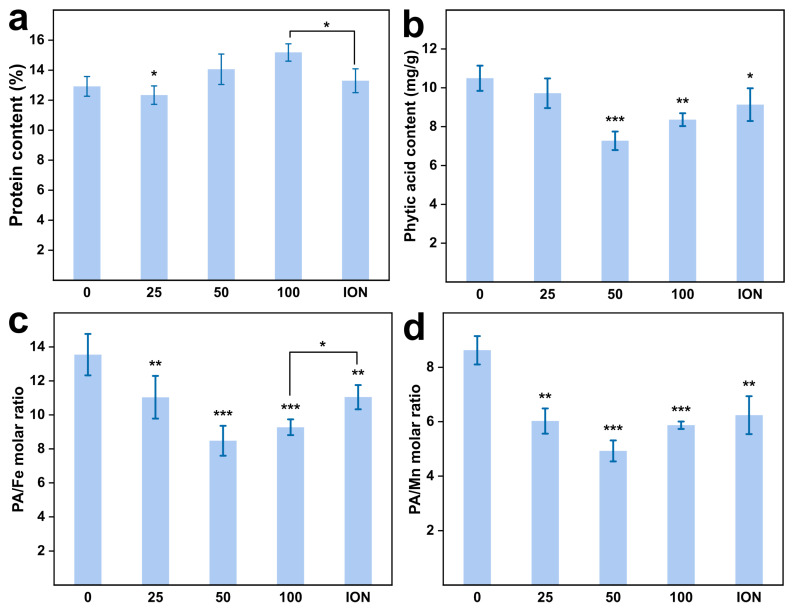
Nutritional quality of wheat grain. (**a**) The crude protein content, (**b**) the phytate content, (**c**) the PA/Fe molar ratio, and (**d**) the PA/Fe molar ratio in the wheat grain. Abbreviations: “0, 25, 50, 100” means the concentration of the MnFe_2_O_4_ NP application (mg/L); ION means the ionic treatments. Asterisk bracket means difference between the 100 mg/L NP treatment and the ionic treatment. * represents statistically significant at *p* < 0.05, ** shows statistically significant at *p* < 0.01, while *** shows statistically significant at *p* < 0.001 between the control and NP treatment.

Protein is one of the key factors influencing the analysis of grain quality. Compared to the ion group, the protein content in the 100 mg/L MnFe_2_O_4_ NP treatment significantly increased (*p* < 0.05), but we did not find significant alterations in the grain protein content when compared to the control (Figure 7a). Besides the absolute concentration of iron in grains, the bioavailability of iron plays a pivotal role in the biofortification process. Phytic acid, within the realm of plant and seed biology, is primarily regarded as a compound responsible for storing P and minerals or as an integral metabolite crucial for maintaining phosphorus homeostasis [44]. However, the polyanionic nature of phytic acid results in its higher affinity for positively charged mineral cations such as iron. This leads to the formation of phytate; these complex insoluble salts significantly reduce the bioavailability of these nutrients to consumers [45].

After the application of iron nano-fertilizer, there was a significant decrease in phytic acid (PA) content, the PA/Fe ratio, and the PA/Mn ratio (Figure 7b–d). As shown in Figure 7, compared to the control, the lowest values were observed at an application concentration of 50 mg/L, with a respective reduction in PA content from 11 ± 0.65 mg/g to 7.3 ± 0.48 mg/g (31%) and 20% reduction in the application concentration of 100 mg/L (Figure 7b). Additionally, the PA/Fe ratios decreased by 37% in the 50 mg/L NP dose from 13.5 ± 0.65 mg/g, and decreased by 32% in the 100 mg/L NP dose from 9.3 ± 0.5 mg/g; the PA/Mn ratios decreased by 43% and 32% in the 50 mg/L and 100 mg/L NP treatments, respectively. This indicates that the application of MnFe_2_O_4_ nano-fertilizer is effective in reducing phytic acid levels. The findings agreed with Lian’s findings that after applying nanoparticles with different particle sizes, the PA decreased significantly [46].

## 3. Materials and Methods

### 3.1. Synthesis and Properties of MnFe_2_O_4_ Nanoparticles

MnFe oxide NPs have been made via a simple co-precipitation approach [22]. Briefly, FeCl_3_·6H_2_O (5.41 g, AR, 99%) and MnCl_2_·4H_2_O (1.98 g, AR, 99%) at the molar ratio of Fe^3+^:Mn^2+^ = 2:1 were added in 100 mL deionized (DI) water and dissolved. Then, the above-mentioned mixture solution added to 3 M NaOH (AR, 95%) solution slowly at 95 °C with vigorous stirring conditions in a water bath. After vigorous stirring for 2 h, the reaction mixture underwent triple washing in deionized water and absolute ethyl alcohol, respectively. After centrifugation at 4000× *g* for 10 min, the resulting powder was dried in an oven at 60 °C for 36 h. The solid product was subsequently finely ground when cooled to room temperature.

Scanning electron microscopy (SEM, G300, Zeiss, Jena, Germany) measurement was performed to identify the surface morphology of NPs. Crystalline structural analysis of the MnFe_2_O_4_ nanocomposite was studied using X-ray powder diffraction (XRD, D8 Advance, Bruker, Ettlingen, Germany). The evaluation of magnetic properties was characterized by vibrating sample magnetometer (VSM, Quantum Design, Inc., San Diego, CA, USA). Elemental and content analysis of nanoparticles was carried out with energy dispersive X-ray spectrometer (EDX, Bruker Nano GmbH, Berlin, Germany).

### 3.2. Field Experiment and Plant Growth

Wheat (Zhengmai 10) seeds were purchased from a seed industry located at the experimental site in China. The experiment was performed under an open field, which is a typical paddy soil area in Huzhou, Zhejiang Province, China, with an average annual temperature of 13–22 °C and annual average precipitation of 1309 mm. A randomized complete block design with three replications per treatment was studied for the experiment. Each micro-field was 10 m^2^ (2 m × 5 m). The basic properties of soil were measured and detailed below: pH 4.92, total organic carbon 26.10 g/kg, total organic nitrogen 2.14 g/kg, CEC 12.08 cmol^+^/kg, TP 528.03 mg/kg. Total Zn, Mn, Fe concentrations of the soil were 88.81 mg/kg, 417 mg/kg, and 27,619.8 mg/kg, respectively. DTPA-Zn was 3.57 mg/kg; DTPA-Mn, 62.67 mg/kg; DTPA-Fe, 269.45 mg/kg.

There were four treatments with different MnFe_2_O_4_ oxide NP doses (0, 25, 50, 100 mg/L), and an ionic control (MnSO_4_·H_2_O and FeSO_4_·7H_2_O) had equivalent Mn and Fe ions to the 100 mg/L nanoparticles. Controlled treatment (0 mg/L) was only sprayed DI water. All the treatments were added with Tween-20 (0.1%) as a surfactant to ensure the penetration into leaves. Sonicated in an ultrasonic bath for 40 min prior to spraying to homogenize the mixture. In the jointing stage (150 d, BBCH-scale 41), the first foliar was applied, and the second spray was in the booting (180 d, BBCH-scale 52), the last spray was in the grain-filling stage (190 d, BBCH-scale 73). Each replicate in this experiment was sprayed with 1 L solution and had three replicates. The basal NPK fertilizers (110, 45, 20 kg/ha, respectively) were applied following local cultivation strategies, and no supplemental irrigation was used during the wheat growing season (from November 2022 to May 2023).

### 3.3. Measurement of Chlorophyll Concentrations

After three days of the last foliar application, the leaf sample was collected to determine the photosynthetic pigments. Briefly, fresh leaf tissue was weighed, chopped, and homogenized in a 15 mL brown tube with 10 mL 95% ethanol solution in the dark for three days. Using a plate reader (Biotek, Synergy Neo2, Winooski, VT, USA), the absorbance values were measured at 655 and 649 wavelengths.

### 3.4. Plant Harvesting and Agronomic Traits

In late May, the wheat grains were harvested, 1 m^2^ area was taken per micro-field and carried out with wheat yield and biomass. Subsequently, each replicate was collected as 100 random wheat plants and separated into different parts for the next analysis. The samples were washed with DI water thoroughly thrice. All the tissues were killed at 105 °C for 15 min and then dried at 65 °C for two days. Finely ground samples were subjected to further analysis: spike weight, thousand-grain weight, grain and biological yield. Fresh samples were kept at −80 °C. The harvest index (HI) plays a vital role in optimizing agricultural practices and enhancing food production, which was calculated as the ratio of the harvested grain and the total aboveground wheat (DW).

### 3.5. Assay of the Protein Quality

The crude protein was determined by Kjeldahl analyzer [47]. Briefly, the grain powder (0.2 g) was mixed with 5 mL H_2_SO_4_, shaken up, then kept overnight. The mixture solution was digested at 180 °C for 10 min, then up to 380 °C for 45 min, cooled to 280 °C, digested for 15 min, and dropped with H_2_O_2_ to make sure the homogenate was clear. After digestion, the final volume was diluted to 50 mL using DI water when the solution was cooled to room temperature. Finally, the concentration of ammonium nitrogen was determined by Kjeldahl analyzer (Kjelflex K-360/K, Buchi, Flawil, Switzerland). The conversion coefficient of nitrogen to protein in wheat grains is 5.83.

### 3.6. Elemental Content and Phytic Acid in Wheat

A high-throughput ball mill (Jxfstprp-24, Jinxin, Shanghai, China) was used to fine the wheat samples before the elemental analysis. About 0.2 g wheat samples were mixed with 4 mL HNO_3_ and 1 mL HClO_4_, kept overnight, and then digested at 180 °C. Acid-digested resultant was diluted up to 50 mL and passed through a 0.45 μm membrane to quantify the element of wheat using the inductively coupled plasma–mass spectrometry ICP-MS (Plasma Quant MS, Analytik Jena, Jena, Germany) analysis, or the inductively coupled plasma–optical emission spectroscopy (ICP-OES) (ICP6000, Thermo Fisher Scientific, Oxford, UK) analysis. Duplicates, blank solutions, and certified standard reference materials were also digested to verify the quality during the experiment. Quality assurance and control were ensured through the use of the standard wheat sample (GBW(E)100495) provided and certified by the China National Institute of Metrology (CRMs), with the recovery rates falling in the range of 90–110%.

The determination of phytate content in wheat grains was measured according to the protocol described by Lian et al. [48]. Briefly, about 1.0 g of grain sample was added to a 20 mL HCL-Na_2_SO_4_ (10% *w*/*v*) for 2 h under continuous agitation at 25 °C and 200 rpm. After centrifugation at 4000× *g* for 20 min at room temperature, the resulting supernatant was collected, mixed with an equal volume of 15% (*w*/*v*) trichloroacetic acid, then stored at 4 °C for 2 h. Subsequent centrifugation at 10,000× *g* for 10 min was carried out and then adjusted to pH 6.5 using 0.75 M NaOH before reacting with ferric chloride-sulfosalicylic acid for 20 min. Lastly, the final solution was measured at 500 nm by a plate reader (Biotek, Synergy Neo2). The potential bioavailability of Fe was estimated by the molar ratio of PA/Fe and PA/Mn in wheat whole grain, which was calculated as follows:

$$PA/Fe\;molar\;ratio=\frac{(PA\;content\;in\;mg\;{kg}^{-1})/660}{(Fe\;content\;in\;mg\;{kg}^{-1})/56}$$

$$PA/Mnmolar\;ratio=\frac{(PA\;content\;in\;mg\;{kg}^{-1})/660}{(Mn\;content\;in\;mg\;{kg}^{-1})/55}$$

### 3.7. Fe Biochemical Stains in Wheat Grains

For the distribution of Fe in wheat grains, we utilized 2% hydrochloric acid and 2% potassium ferrocyanide reagent to assess the localization of Fe in wheat grains. Perls’ solution should be prepared freshly. The mixture solution was used as a staining buffer to stain the grain, which was cut in half [49].

## 4. Statistical Analysis

Experimental data were reported as the mean ± SD of three replicates. One-way analysis of variance (ANOVA) and a least significant difference (LSD) test were conducted to obtain *p*-values (*p* < 0.05). In the group treated by MnFe_2_O_4_ NPs (from 25 to 100 mg/L), comparisons were made solely with the control (0 mg/L). An independent samples *t*-test was employed to analyze the differences between the treatment group (spraying 100 mg/L of MnFe_2_O_4_ NPs) and the ion group. The normality and homogeneity of variances were confirmed through Shapiro–Wilk and Levene’s tests. Non-parametric independent sample tests were used for the S concentration of the straw and glume. The mean values plus standard deviations (SD) for each figure are provided in the Supplementary Materials. All analyses were carried out using IBM SPSS Statistics software (Version 27.0, Armonk, NY, USA).

## 5. Conclusions

This study aimed to investigate the impact of NP (nanoparticles of synthetic iron) spraying on various tissue components of wheat throughout its growth cycle through field experiments. The experiment revealed that the application of synthesized nano-iron significantly increased (*p* < 0.05) the Fe (from 66 ± 2.3 to 77 ± 2.7 mg/kg) and Mn (from 101 ± 1.2 to 134 ± 1.6 mg/kg) content in wheat grains. Moreover, the Fe and Mn content in other plant tissues, such as straw, also exhibited a substantial increase (*p* < 0.05). The levels of other elements, including Mg and Ca, showed an upward trend in various plant tissues, promoting the growth and development of wheat while effectively reducing the phytic acid content in wheat grains. In terms of grain yield and biological yield, there were no significant differences between the 100 mg/L treatment group and the ion group. However, there was a significant increase in Mn, Ca, and Zn content in the glume, as well as a significant increase in Cu, Mg, and S concentration in the straw. This provides a nutritional source for straw return. Compared to the ion treatment, the 100 mg/L NP treatments can significantly increase the crude protein content in the wheat grain, and the NP treatments can significantly reduce the PA/Fe, thereby enhancing the bioavailability of Fe. At the same time, according to the results, when applying the 50 mg/L NPs, the effect may already be superior to that of the ion group. However, uncertainties remain regarding the translocation and absorption mechanisms of NPs in plants after the complete growth cycle. It is crucial to further investigate whether the application of nanomaterials in the form of sprays could lead to accumulation in the soil, potentially affecting the growth of subsequent crop generations.

## Data Availability

This manuscript does not report data generation or analysis. Data are available from the authors upon request.

## Supplementary Materials

The following supporting information can be downloaded at: https://www.mdpi.com/article/10.3390/plants13101395/s1, Figure S1: The content of pigment; Table S1: Agronomic parameters of wheat; Table S2: Fe concentration in different wheat tissues; Table S3: Mn concentration in different wheat tissues; Table S4: Ca concentration in different wheat tissues; Table S5: Mg concentration in different wheat tissues; Table S6: Cu concentration in different wheat tissues; Table S7: Zn concentration in different wheat tissues; Table S8: P concentration in different wheat tissues; Table S9: S concentration in different wheat tissues; Table S10: Nutritional quality of wheat grain; Table S11: The content of pigment.

## References

[1] Jiang X.-L., Tian J.-C., Zhi H., Zhang W.-D. (2008). Protein content and amino acid composition in grains of wheat-related species. Agric. Sci. China.

[2] Kong D., Khan S.A., Wu H., Liu Y., Ling H.Q. (2022). Biofortification of iron and zinc in rice and wheat. J. Integr. Plant Biol..

[3] Bouis H.E., Hotz C., McClafferty B., Meenakshi J., Pfeiffer W.H. (2011). Biofortification: A new tool to reduce micronutrient malnutrition. Food Nutr. Bull..

[4] World Health Organization (2017). Malnutrition: It’s about more than Hunger. https://www.who.int/news-room/commentaries/detail/malnutrition-it-s-about-more-than-hunger.

[5] World Health Organization (2021). Levels and Trends in Child Malnutrition: UNICEF.

[6] Lowe N.M. (2021). The global challenge of hidden hunger: Perspectives from the field. Proc. Nutr. Soc..

[7] Ghormade V., Deshpande M.V., Paknikar K.M. (2011). Perspectives for nano-biotechnology enabled protection and nutrition of plants. Biotechnol. Adv..

[8] Gilbertson L.M., Pourzahedi L., Laughton S., Gao X., Zimmerman J.B., Theis T.L., Westerhoff P., Lowry G.V. (2020). Guiding the design space for nanotechnology to advance sustainable crop production. Nat. Nanotechnol..

[9] Arora S., Murmu G., Mukherjee K., Saha S., Maity D. (2022). A comprehensive overview of nanotechnology in sustainable agriculture. J. Biotechnol..

[10] Lv X., Sha H., Ye Z., Wang Y., Mao B. (2023). Nanomaterials in plant management: Functions, mechanisms and prospects. Environ. Sci. Nano.

[11] Kah M., Kookana R.S., Gogos A., Bucheli T.D. (2018). A critical evaluation of nanopesticides and nanofertilizers against their conventional analogues. Nat. Nanotechnol..

[12] Su Y., Ashworth V., Kim C., Adeleye A.S., Rolshausen P., Roper C., White J., Jassby D. (2019). Delivery, uptake, fate, and transport of engineered nanoparticles in plants: A critical review and data analysis. Environ. Sci. Nano.

[13] Song W., Zhao B., Wang C., Ozaki Y., Lu X. (2019). Functional nanomaterials with unique enzyme-like characteristics for sensing applications. J. Mater. Chem. B.

[14] Tombuloglu H., Tombuloglu G., Slimani Y., Ercan I., Sozeri H., Baykal A. (2018). Impact of manganese ferrite (MnFe_2_O_4_) nanoparticles on growth and magnetic character of barley (*Hordeum vulgare* L.). Environ. Pollut..

[15] Giust D., Lucío M.I., El-Sagheer A.H., Brown T., Williams L.E., Muskens O.L., Kanaras A.G. (2018). Graphene oxide–upconversion nanoparticle based portable sensors for assessing nutritional deficiencies in crops. ACS Nano.

[16] Lowry G.V., Avellan A., Gilbertson L.M. (2019). Opportunities and challenges for nanotechnology in the agri-tech revolution. Nat. Nanotechnol..

[17] Li P., Wang A., Du W., Mao L., Wei Z., Wang S., Yuan H., Ji R., Zhao L. (2020). Insight into the interaction between Fe-based nanomaterials and maize (*Zea mays*) plants at metabolic level. Sci. Total Environ..

[18] Lin D., Xing B. (2008). Root uptake and phytotoxicity of ZnO nanoparticles. Environ. Sci. Technol..

[19] Avellan A., Yun J., Zhang Y., Spielman-Sun E., Unrine J.M., Thieme J., Li J., Lombi E., Bland G., Lowry G.V. (2019). Nanoparticle size and coating chemistry control foliar uptake pathways, translocation, and leaf-to-rhizosphere transport in wheat. ACS Nano.

[20] Hong J., Wang C., Wagner D.C., Gardea-Torresdey J.L., He F., Rico C.M. (2021). Foliar application of nanoparticles: Mechanisms of absorption, transfer, and multiple impacts. Environ. Sci. Nano.

[21] Dimkpa C.O. (2018). Soil properties influence the response of terrestrial plants to metallic nanoparticles exposure. Curr. Opin. Environ. Sci. Health.

[22] Asli S., Neumann P.M. (2009). Colloidal suspensions of clay or titanium dioxide nanoparticles can inhibit leaf growth and transpiration via physical effects on root water transport. Plant Cell Environ..

[23] Marchiol L., Iafisco M., Fellet G., Adamiano A. (2020). Nanotechnology support the next agricultural revolution: Perspectives to enhancement of nutrient use efficiency. Adv. Agron..

[24] Dimkpa C.O., Singh U., Adisa I.O., Bindraban P.S., Elmer W.H., Gardea-Torresdey J.L., White J.C. (2018). Effects of manganese nanoparticle exposure on nutrient acquisition in wheat (*Triticum aestivum* L.). Agronomy.

[25] Zhu Y., Zhang Q., Li Y., Pan Z., Liu C., Lin D., Gao J., Tang Z., Li Z., Wang R. (2023). Role of Soil and Foliar-Applied Carbon Dots in Plant Iron Biofortification and Cadmium Mitigation by Triggering Opposite Iron Signaling in Roots. Small.

[26] Salehi H., Chehregani A., Lucini L., Majd A., Gholami M. (2018). Morphological, proteomic and metabolomic insight into the effect of cerium dioxide nanoparticles to Phaseolus vulgaris L. under soil or foliar application. Sci. Total Environ..

[27] Ramírez-Rodríguez G.B., Miguel-Rojas C., Montanha G.S., Carmona F.J., Dal Sasso G., Sillero J.C., Skov Pedersen J., Masciocchi N., Guagliardi A., Pérez-de-Luque A. (2020). Reducing nitrogen dosage in Triticum durum plants with urea-doped nanofertilizers. Nanomaterials.

[28] Ramírez-Rodríguez G.B., Dal Sasso G., Carmona F.J., Miguel-Rojas C., Pérez-de-Luque A., Masciocchi N., Guagliardi A., Delgado-López J.M. (2020). Engineering biomimetic calcium phosphate nanoparticles: A green synthesis of slow-release multinutrient (NPK) nanofertilizers. ACS Appl. Bio Mater..

[29] Gao X., Avellan A., Laughton S., Vaidya R., Rodrigues S.M., Casman E.A., Lowry G.V. (2018). CuO nanoparticle dissolution and toxicity to wheat (*Triticum aestivum*) in rhizosphere soil. Environ. Sci. Technol..

[30] Hu J., Guo H., Li J., Gan Q., Wang Y., Xing B. (2017). Comparative impacts of iron oxide nanoparticles and ferric ions on the growth of Citrus maxima. Environ. Pollut..

[31] Elhaj Baddar Z., Unrine J.M. (2021). Effects of Soil pH and Coatings on the Efficacy of Polymer coated ZnO Nanoparticulate fertilizers in Wheat (*Triticum aestivum*). Environ. Sci. Technol..

[32] Yue L., Feng Y., Ma C., Wang C., Chen F., Cao X., Wang J., White J.C., Wang Z., Xing B. (2022). Molecular mechanisms of early flowering in tomatoes induced by manganese ferrite (MnFe_2_O_4_) nanomaterials. ACS Nano.

[33] Parsons J.G., Lopez M.L., Peralta-Videa J.R., Gardea-Torresdey J.L. (2009). Determination of arsenic (III) and arsenic (V) binding to microwave assisted hydrothermal synthetically prepared Fe_3_O_4_, Mn_3_O_4_, and MnFe_2_O_4_ nanoadsorbents. Microchem. J..

[34] Akhlaghi N., Najafpour-Darzi G. (2021). Manganese ferrite (MnFe_2_O_4_) Nanoparticles: From synthesis to application—A review. J. Ind. Eng. Chem..

[35] Kwon J., Kim J.-H., Kang S.-H., Choi C.-J., Rajesh J.A., Ahn K.-S. (2017). Facile hydrothermal synthesis of cubic spinel AB2O4 type MnFe_2_O_4_ nanocrystallites and their electrochemical performance. Appl. Surf. Sci..

[36] Noor R., Yasmin H., Ilyas N., Nosheen A., Hassan M.N., Mumtaz S., Khan N., Ahmad A., Ahmad P. (2022). Comparative analysis of iron oxide nanoparticles synthesized from ginger (*Zingiber officinale*) and cumin seeds (*Cuminum cyminum*) to induce resistance in wheat against drought stress. Chemosphere.

[37] Huang X., Keller A.A. (2021). Metabolomic response of early-stage wheat (*Triticum aestivum*) to surfactant-aided foliar application of copper hydroxide and molybdenum trioxide nanoparticles. Nanomaterials.

[38] Li M., Zhang P., Adeel M., Guo Z., Chetwynd A.J., Ma C., Bai T., Hao Y., Rui Y. (2021). Physiological impacts of zero valent iron, Fe_3_O_4_ and Fe_2_O_3_ nanoparticles in rice plants and their potential as Fe fertilizers. Environ. Pollut..

[39] Li R., He J., Xie H., Wang W., Bose S.K., Sun Y., Hu J., Yin H. (2019). Effects of chitosan nanoparticles on seed germination and seedling growth of wheat (*Triticum aestivum* L.). Int. J. Biol. Macromol..

[40] Parry M.A., Reynolds M., Salvucci M.E., Raines C., Andralojc P.J., Zhu X.G., Price G.D., Condon A.G., Furbank R.T. (2011). Increasing photosynthetic capacity and efficiency. J. Exp. Bot..

[41] Dwivedi A.D., Yoon H., Singh J.P., Chae K.H., Rho S.-c., Hwang D.S., Chang Y.-S. (2018). Uptake, distribution, and transformation of zerovalent iron nanoparticles in the edible plant Cucumis sativus. Environ. Sci. Technol..

[42] Iannone M.F., Groppa M.D., Zawoznik M.S., Coral D.F., van Raap M.B.F., Benavides M.P. (2021). Magnetite nanoparticles coated with citric acid are not phytotoxic and stimulate soybean and alfalfa growth. Ecotoxicol. Environ. Saf..

[43] Lian J., Cheng L., Zhai X., Wu R., Liu W., Pan J., Shohag M., Xin X., He Z., Yang X. (2022). Foliar spray of combined metal-oxide nanoparticles alters the accumulation, translocation and health risk of Cd in wheat (*Triticum aestivum* L.). J. Hazard. Mater..

[44] Raboy V., Gerbasi P.F., Young K.A., Stoneberg S.D., Pickett S.G., Bauman A.T., Murthy P.P., Sheridan W.F., Ertl D.S. (2000). Origin and seed phenotype of maize low phytic acid 1-1 and low phytic acid 2-1. Plant Physiol..

[45] Angel R., Tamim N., Applegate T., Dhandu A., Ellestad L. (2002). Phytic acid chemistry: Influence on phytin-phosphorus availability and phytase efficacy. J. Appl. Poult. Res..

[46] Lian J., Cheng L., Wang X., Chen Y., Deng C., Wang Y., Pan J., Shohag M.J.I., Xin X., He Z. (2024). Bespoke ZnO NPs Synthesis Platform to Optimize Their Performance for Improving the Grain Yield, Zinc Biofortification, and Cd Mitigation in Wheat. ACS Sustain. Chem. Eng..

[47] Kirk P.L. (1950). Kjeldahl method for total nitrogen. Anal. Chem..

[48] Lian J., Cheng L., Zhai X., Wu R., Huang X., Chen D., Pan J., Shohag M., Xin X., Ren X. (2023). Zinc glycerolate (Glyzinc): A novel foliar fertilizer for zinc biofortification and cadmium reduction in wheat (*Triticum aestivum* L.). Food Chem..

[49] Velu G., Bhattacharjee R., Rai K.N., Sahrawat K., Longvah T. (2008). A simple and rapid screening method for grain zinc content in pearl millet. J. SAT Agric. Res..

