# Supplementary material for: Nanotechnology in Agriculture: Manganese Ferrite Nanoparticles as a Micronutrient Fertilizer for Wheat
Source: Plants (Basel). 2024 May 17;13(10):1395. doi: 10.3390/plants13101395 (PMC11124989; doi:10.3390/plants13101395)
Supplement: Supplementary file 1 [file plants-13-01395-s001.zip › plants-2984371-supplementary.pdf]

Supporting Information

Nanotechnology in agriculture: Manganese ferrite nanoparticles as a micronutrient fertilizer for wheat

Xiwei Huang [a], Xin Wang [a], Xingxing Liu [a] , Liping Cheng [a] , Jianqing Pan [b], Xiaoe Yang [a,*]

[a] Ministry of Education (MOE) Key Laboratory of Environmental Remediation and Ecosystem Health, College of Environmental and Resources Sciences, Zhejiang University, Hangzhou, 310058, China

[b] Agriculture Bureau of Changxing County, Zhejiang Province, Huzhou, 323000, China

[*]**Corresponding Author:** Xiaoe Yang

E-mail: xeyang@zju.edu.cn (X.E. Yang)

Full Postal Address: No. 866 Yuhangtang Road, College of Environmental and Resources Sciences, Zhejiang University, Hangzhou, 310058, China

Phone/Fax: +86 0571 88982907.

Figure S1. The content of pigment. "0, 25, 50, 100" means the concentration of the $MnFe_2O_4$ NPs application (mg/L); ION means the ionic treatments. * represents the statistically significant at $p < 0.05$, ** shows the statistically significant at $p < 0.01$ while *** shows the statistically significant at $p < 0.001$ between the control and NPs treatments.

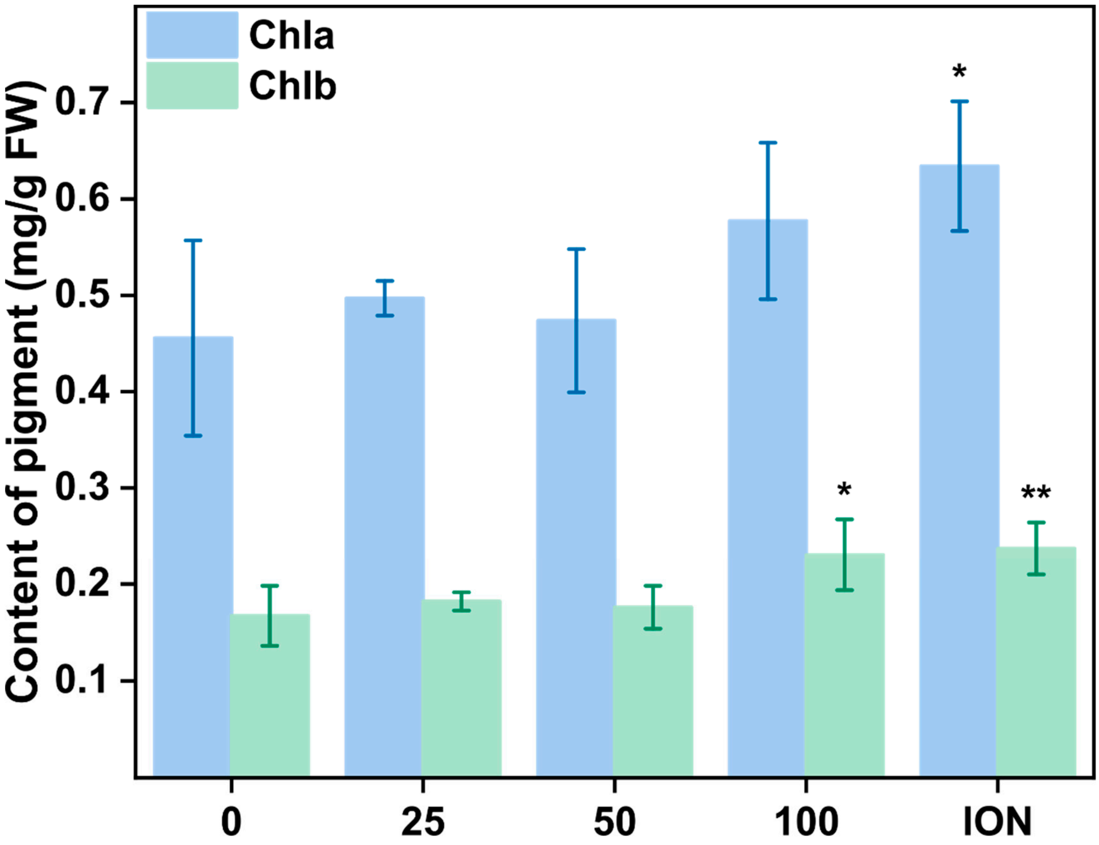

Table S1. Agronomic parameters of wheat. (n = 3; Mean ± SD). "0, 25, 50, 100" means the concentration of the $MnFe_2O_4$ NPs application (mg/L); ION means the ionic treatments. * represents the statistically significant at $p < 0.05$, ** shows the statistically significant at $p < 0.01$ while *** shows the statistically significant at $p < 0.001$ between the control and NPs treatments.

treatment	Spike weight (g)	Grain number per spike	Spike weight (g)	Grain number per spike	Spike weight (g)	Grain number per spike
0	1.7±0.16	38.4±0.7	37.5±1.16	4.2±0.4	10.7±0.8	0.43±0.005
25	1.7±0.24	31.4±1.8**	39.1±0.37	3.7±0.27*	10.9±0.39	0.38±0.053
50	2±0.03*	41.8±1.1	39.2±0.21	5±0.12**	10.9±0.28	0.48±0.005***
100	2±0.07	43.6±4.2*	36.2±4.74	4.9±0.19**	11±0.95	0.46±0.01**
ION	1.9±0.09	41.8±2.1	39±0.24	4.7±0.26*	10.8±0.05	0.44±0.02

Table S2. Fe concentration in different wheat tissues (mg/kg) (n = 3; Mean ± SD) "0, 25, 50, 100" means the concentration of the MnFe2O4 NPs application (mg/L); ION means the ionic treatments. * represents the statistically significant at p < 0.05, **

shows the statistically significant at $p < 0.01$ while *** shows the statistically significant at $p < 0.001$ between the control and NPs treatments.

Treatment	grain	glume	straw	root
0	65.9±2.3	255.5±16	126.3±30	3220.4±596
25	74.9±3.2**	313.6±9**	172.1±17*	2803.3±331
50	73±3.0*	299.9±17**	162.4±15	4225.3±131*
100	76.5±2.7**	310.5±27**	170.4±19*	3690.2±548
ION	70.1±3.3	304.6±10**	146.3±25	3681.4±768

Table S3. Mn concentration in different wheat tissues (mg/kg) (n = 3; Mean ± SD). "0, 25, 50, 100" means the concentration of the MnFe2O4 NPs application (mg/L); ION means the ionic treatments. * represents the statistically significant at $p < 0.05$, ** shows the statistically significant at $p < 0.01$ while *** shows the statistically significant at $p < 0.001$ between the control and NPs treatments.

Treatment	grain	glume	straw	root
0	101.3±1.2	105.8±5.8	161.6±16	40.1±2.4
25	134.4±1.6***	106.9±1.1	210.3±7	45.7±3.2
50	123.1±1.6***	146.6±14.5**	242.7±37**	45±1.7**
100	118.7±2.8***	140.4±4.9*	191.1±9	46.1±9.9
ION	122.2±2.6***	122.8±5.5	225.7±7**	42.2±2.5

Table S4. Ca concentration in different wheat tissues (g/kg) (n = 3; Mean ± SD). "0, 25, 50, 100" means the concentration of the MnFe2O4 NPs application (mg/L); ION means the ionic treatments. * represents the statistically significant at $p < 0.05$, ** shows the statistically significant at $p < 0.01$ while *** shows the statistically significant at $p < 0.001$ between the control and NPs treatments.

Treatment	Grain	Glume	Straw
0	0.26±0.02	0.78±0.05	2.05±0.18
25	0.36±0.02***	0.83±0.03	2.1±0.26
50	0.43±0.03***	0.94±0.05***	2.47±0.21*
100	0.32±0.01*	1.07±0.03***	2.68±0.26**
ION	0.29±0.04	0.96±0.02***	2.22±0.17

Table S5. Mg concentration in different wheat tissues (g/kg) (n = 3; Mean ± SD). "0, 25, 50, 100" means the concentration of the MnFe2O4 NPs application (mg/L); ION means the ionic treatments. * represents the statistically significant at $p < 0.05$, ** shows the statistically significant at $p < 0.01$ while *** shows the statistically significant at $p < 0.001$ between the control and NPs treatments.

Treatment	Grain	Glume	Straw
0	0.26±0.02	0.78±0.05	2.05±0.18
25	0.36±0.02***	0.83±0.03	2.1±0.26
50	0.43±0.03***	0.94±0.05***	2.47±0.21*
100	0.32±0.01*	1.07±0.03***	2.68±0.26**
ION	0.29±0.04	0.96±0.02***	2.22±0.17

Table S6. Cu concentration in different wheat tissues (g/kg) (n = 3; Mean ± SD). "0, 25, 50, 100" means the concentration of the MnFe2O4 NPs application (mg/L); ION means the ionic treatments. * represents the statistically significant at $p < 0.05$, ** shows the statistically significant at $p < 0.01$ while *** shows the statistically significant at $p < 0.001$ between the control and NPs treatments.

Treatment	Grain	Glume	Straw
0	8.4±0.33	2.57±0.09	1.59±0.08
25	8.02±0.33	2.58±0.39	1.71±0.11
50	8.33±0.84	3.23±0.07**	2.12±0.11***
100	8.04±0.47	2.73±0.14	1.94±0.09**
ION	8.6±0.15	2.71±0.62	1.56±0.19

Table S7. Zn concentration in different wheat tissues (n = 3; Mean ± SD). "0, 25, 50, 100" means the concentration of the $MnFe_2O_4$ NPs application (mg/L); ION means the ionic treatments. * represents the statistically significant at $p < 0.05$, ** shows the statistically significant at $p < 0.01$ while *** shows the statistically significant at $p < 0.001$ between the control and NPs treatments.

Treatment	Grain	Glume	Straw
0	59.4±1.1	27.2±1.4	10.4±1.3
25	55.9±2.2	24.7±1.4	12.5±1.4
50	64.7±5.1	27.1±1.4	12.4±3.7
100	56.7±3.4	21.9±1.5**	11.6±0.9
ION	54.6±3.5	27.9±3.1	10.7±0.6

Table S8. P concentration in different wheat tissues (g/kg) (n = 3; Mean ± SD).. "0, 25, 50, 100" means the concentration of the $MnFe_2O_4$ NPs application (mg/L); ION means the ionic treatments. * represents the statistically significant at $p < 0.05$, ** shows the statistically significant at $p < 0.01$ while *** shows the statistically significant at $p < 0.001$ between the control and NPs treatments.

Treatment	Grain	Glume	Straw
0	4.16±0.15	0.27±0.02	0.24±0.01
25	4.5±0.1**	0.26±0.06	0.23±0.02
50	4.27±0.15	0.27±0.08	0.22±0.03
100	4.28±0.08	0.31±0.02	0.27±0.01*
ION	4.36±0.06	0.33±0.11	0.3±0.02**

Table S9. S concentration in different wheat tissues (g/kg) (n = 3; Mean ± SD). "0, 25, 50, 100" means the concentration of the $MnFe_2O_4$ NPs application (mg/L); ION means the ionic treatments. * represents the statistically significant at $p < 0.05$, ** shows the statistically significant at $p < 0.01$ while *** shows the statistically significant at $p < 0.001$ between the control and NPs treatments.

Treatment	Grain	Glume	Straw
0	2.11±0.06	0.48±0.03	1.46±0.03
25	2.13±0.07	0.44±0.06	0.89±0.07***
50	2.28±0.15*	0.54±0.05	1.64±0.14
100	2.45±0.07***	0.56±0.01*	1.75±0.05**
ION	2.23±0.03	0.53±0.05	1.47±0.1

Table S10. Nutritional quality of wheat grain (n = 3; Mean ± SD). "0, 25, 50, 100" means the concentration of the $MnFe_2O_4$ NPs application (mg/L); ION means the ionic treatments. * represents the statistically significant at $p < 0.05$, ** shows the statistically significant at $p < 0.01$ while *** shows the statistically significant at $p < 0.001$ between the control and NPs treatments.

Treatment	Protein(%)	PA (mg/kg)	PA/Fe	PA/Mn
0	12.9±0.66	10.5±0.65	13.5±1.2	8.6±0.52
25	12.3±0.61*	9.7±0.76	11±1.3**	6±0.46**
50	14.1±1.01	7.3±0.48***	8.5±0.9***	4.9±0.38***
100	15.2±0.58	8.4±0.33**	9.3±0.5***	5.9±0.14***
ION	13.3±0.79	9.1±0.84*	11±0.7**	6.2±0.7**

Table S11. The content of pigment (mg/kg) (n = 3; Mean ± SD). "0, 25, 50, 100" means the concentration of the $MnFe_2O_4$ NPs application (mg/L); ION means the ionic treatments. * represents the statistically significant at $p < 0.05$, ** shows the statistically significant at $p < 0.01$ while *** shows the statistically significant at $p < 0.001$ between the control and NPs treatments.

Treatment	CHla	CHlb
0	0.46±0.1	0.17±0.03
25	0.5±0.02	0.18±0.01
50	0.47±0.07	0.18±0.02
100	0.58±0.08	0.23±0.04*
ION	0.63±0.07*	0.24±0.03*
